# Evaluating a co-developed pet robot intervention and implementation for residents with dementia in long-term care

**DOI:** 10.3389/frdem.2026.1791588

**Published:** 2026-04-17

**Authors:** Wei Qi Koh, Tracy Carroll, Letetia Gimblett, Ellen Murphy, Nicole Gavin

**Affiliations:** 1 School of Health and Rehabilitation Sciences, The University of Queensland, Brisbane, QLD, Australia; 2 Cooinda House, Specialised Aged Care, Community and Oral Health, Metro North Health, Kippa-Ring, QLD, Australia; 3 Community and Oral Health, Metro North Health, Brighton, QLD, Australia; 4 School of Nursing, Queensland University of Technology, Brisbane, QLD, Australia

**Keywords:** dementia, implementation, nursing home, residential care, robot pets, technology

## Abstract

**Background:**

Pet robots have the potential to improve the wellbeing of people living with dementia, particularly in residential care contexts. Despite increasing interest in their use and a body of research investigating their impact and effectiveness, more research is needed to support their translation into dementia care practice. This paper reports on the co-development and evaluation of a pet robot intervention and its implementation in a residential aged care facility to support the psychosocial wellbeing of residents living with dementia.

**Methods:**

A pet robot intervention protocol and implementation strategies were co-developed in consultation with key stakeholders from a residential aged care facility. Purposive sampling was used to recruit participants (residents) living with dementia. The pet robot intervention was delivered up to three times a week, for up to 30 min each time over a 6-week period. Following the intervention, residents with dementia, their family members and staff involved in delivering the intervention were invited to participate in a semi-structured interview to understand their experiences and perceptions of the impact of the intervention and its implementation. Interviews were audio-recorded, transcribed and analysed using content analysis.

**Results:**

Eight residents with dementia had consented to participate, however one had passed away before the intervention commenced. At the end of the intervention period, eight participants including five residents, one family member and two staff consented to participate in the semi-structured interviews. Four key themes were generated: (1) perceptions of the attributes of pet robots, (2) opening doors: facilitating connections and participation, (3) integrating pet robots into routine care, and (4) moving forward: maximising impact and sustainability.

**Conclusion:**

This study demonstrates the feasibility and potential impact of a 6-week pet robot intervention and implementation in a RACF facility to support residents living with dementia. Findings provide insights into strategies that might increase the routinisation of pet robots in dementia care; future studies could consider further refining, enhancing and formally evaluating these strategies.

## Introduction

It is estimated that at least half of the residents living in residential aged care facilities (RACF) in Australia have dementia (Australian Institute of Health and Welfare, 2025). People with dementia living in residential aged care facilities are especially susceptible to reduced social health as compared to community dwellers (Olsen et al., 2016) and experience reduced opportunities to engage in meaningful activities (Han et al., 2016; Kielsgaard et al., 2021). Interactions with animals have positively impacted the wellbeing of people living with dementia (Lai et al., 2019), providing opportunities for individuals to engage in meaningful activities. Allen et al. (2000) investigated the experience of pet ownership for individuals living with chronic diseases and found that engaging in the occupation of pet care involved holding, hugging, feeding their pet, and adjusting their daily routine to accommodate their care and maintenance. This was often an impetus for them to engage in other activities, such as physical exercise and social events. However, those living in RACFs often have limited access to pets. Most residential aged care facilities do not permit pet ownership, and interactions with pets can raise concerns about adverse effects such as transmission of zoonotic diseases and compromised animal welfare (Lai et al., 2019).

Pet robots are robots that look and behave like animals, and they are innovative technology-based substitutes for live animals (Petersen et al., 2016). The use of pet robots in dementia care have been likened to doll therapy (Koh et al., 2022a), which has been used to provide opportunities for people with dementia to engage in a range of activities to alleviate stress, promote wellbeing and reduce behavioural symptoms (Martín-García et al., 2022). A study by Pike et al. (2021) found that people with dementia had wanted to feed and stroke the robotic cat, and the desire to care for it provided a sense of purpose and reduced behavioural symptoms. Similar findings were reported by the family members in another study by Koh et al. (2022b). This suggests that pet robots should have the potential to support meaningful participation and promote the quality of life among people living with dementia (Kristensen, 2024). The most well-researched pet robot is PARO, a robotic seal which demonstrated positive impacts on the psychosocial health of older people with dementia as demonstrated in several research trials (Bemelmans et al., 2015; Jøranson et al., 2015; Moyle et al., 2017). However its cost (approximately $8,000 AUD) has been prohibitive to purchase outside of the research setting and for individuals (Hung et al., 2019). Other issues relating to the use of PARO, such as infection control and staff workload have consequently emerged (Hung et al., 2019). Lower cost pet robots such as the Joy for All (JfA) cat and dog (approximately $250–300 AUD) demonstrated similar impacts and have emerged as promising alternatives (Koh et al., 2021; Koh et al., 2022b). For instance, a cluster randomised controlled trial by Bradwell et al. (2022) found that these robotic pets led to improvements in neuropsychiatric symptoms among residents in care homes. However, more research is necessary to confirm their effectiveness. Furthermore, most existing studies do not sufficiently consider users’ preferences or explicitly tailor interventions to their needs (Koh et al., 2022b). For instance, users have not been given the option to choose their preferred pet robot design (Bradwell et al., 2019). Considering that the needs, preferences and manifestation of symptoms differ across individuals with dementia, it is possible that tailoring interventions could lead to better outcomes.

### The research-to-implementation gap

While pet robots have been developed and researched in various countries over the last two decades, it is essential to ensure that the research on pet robots is successfully translated in dementia care (Sekhon et al., 2022). As an ethnographic study by Felding et al. (2024) highlighted, pet robots that are acquired by aged care facilities have ended up being abandoned or shelved in storage. It is necessary to investigate how pet robot-based interventions can be integrated into dementia care in a sustainable manner, taking contextual factors into account. Fernandes et al. (2025) conducted a recent scoping review of 42 articles to examine barriers and facilitators to implementing pet robots in long-term care settings. Implementation barriers included concerns about infection control and cleaning, ethical concerns, difficulties ascertaining individuals who might benefit from robotic pets, lack of knowledge on how to introduce robotic pets, resource constraints, and a lack of understanding about the benefits of robotic pets. In contrast, implementation facilitators included seeing the benefits of robotic pets and corresponding impact on staff members’ work routines, training and education, and staff members’ enthusiasm (Fernandes et al., 2025). Koh et al. (2023) conducted an international consensus study involving 56 care professionals and organisational leaders from aged care, and researchers with expertise in psychosocial interventions, dementia and/or implementation science, to identify the most critical and important strategies to integrate pet robots in dementia care. Eleven strategies were identified, and some of these strategies included assessing readiness and identifying barriers and facilitators, and obtaining and using residents’ and their family’s feedback. These strategies need to be further operationalised to ensure contextual relevance to the implementing organisation. According to Pfadenhauer et al. (2015, p.104), context can be defined as ‘a set of circumstances and characteristics that surround the implementation effort’. Context is salient to implementation and should be thought of as being more than just a “backdrop” for implementation (Damschroder et al., 2009), encompassing organisational support, financial resources, social relations and support, leadership, organisational culture and climate, readiness to change, structures, patients, wider environment, feedback, time availability and the physical environment (Nilsen and Bernhardsson, 2019). To best support the integration of pet robots in dementia care, it is necessary to understand the implementation context when considering or operationalising implementation strategies. Based on this, we co-developed a pet robot intervention and implementation program with a residential aged care facility (RACF). Implementation strategies were contextualised in collaboration with the facility staff, and the impact of this program was evaluated.

## Methods

This exploratory study received ethics approval from the ethics review committees at Metro North Health and the University of Queensland (HREC/2024/MNHB/109048).

### Setting and participants

This study took place in a government funded RACF in Southeast Queensland. The facility accommodates up to 60 residents and provides specialised care for people living with dementia. Purposive sampling was used to recruit residents with dementia from the RACF between January to March 2025. Table 1 shows the inclusion and exclusion criteria:

**Table 1:** Inclusion and exclusion criteria.

Participants	Inclusion critiera	Exclusion criteria
Residents with dementia	60 years and above Understand and/or speak English Have a formal or probable diagnosis of dementia Living in the care facility for at least one month	Not likely to be residing in the facility for the study duration Medically unfit (e.g., acute illness, require frequent admission to a hospital, or aged below 60)
Care staff	Delivered the pet robot-based intervention, or Provided care to the resident with dementia during the intervention	Not delivering or providing care to the resident
Family members or friends	Visit the resident at least three times during the 6-week intervention period	Have not visited the resident, or visited the resident less than three times during 6-week intervention period

Staff members identified prospective residents who met the inclusion criteria. A staff member (LG) first approached the residents and their family members to ascertain their interest in participation. If the residents and/or their family members were amenable to consider participation, permission was sought for the lead researcher (WQK) to contact the resident and/or the family member to provide more information and to obtain written consent as appropriate.

### Intervention and implementation

Pet robots used in this research were the Joy for All (JfA) companion robots developed by Ageless Innovation (https://joyforall.com/), which have touch sensors and movement sensors to provide interactive responses, such as meowing and rolling over (cat), barking (dog) or chirping (bird).

### Selecting and contextualising implementation strategies

The Nursing Director of the RACF identified five key stakeholders, who were staff members including nursing staff and recreational officers (also known as activity coordinators or diversional therapists). These stakeholders took part in three monthly meetings (60–90 min/meeting) between February to May 2024. Given that assessing implementation readiness has been identified as the most salient strategy for implementing pet robots (Koh et al., 2023), the initial meeting focused on establishing readiness and to identify implementation barriers and facilitators. Topics discussed focused on understanding existing programs and activities for residents with dementia, and whether staff perceived a role and need for robotic pets. These meetings revealed organisational readiness to adopt pet robots as a prospective intervention within the facility (Koh et al., 2023). Subsequent discussions enabled an understanding of the organisational context such as the physical environment, resources, staff schedules and availability. Thereafter, discussions centred around implementation re-examination, and when and how residents and their family members’ feedback could be sought. In a fourth meeting in November 2024, stakeholders established the importance of an additional strategy to conduct educational meetings, so that all staff members are informed about the pet robot intervention. Topics should include providing evidence for the use of robotic pets in dementia care, and their impact on caregiving. The educational meetings should also provide all staff with the opportunity to ask questions and discuss any concerns that they may have.

### Co-developing the intervention protocol

Based on staff feedback, five JfA companion robotic pets were purchased, this included two different colours of cats and dogs, and a bird to provide residents with the opportunity to select a robot based on their personal preferences. A review of the literature on pet robots (Pu et al., 2020; Lu et al., 2021), doll therapy (Martín-García et al., 2022) was undertaken to ascertain suitable intervention frequency; specifically, two systematic reviews (Pu et al., 2020; Lu et al., 2021) revealed that robotic pets have typically been delivered for between 15 and 45 min per session, two to three times weekly, for a period of between 5 and 12 weeks. This led to the development of an initial intervention protocol that was presented to the key stakeholders, along with current research evidence. Staff were asked to consider research evidence and propose revisions to the intervention protocol (e.g., frequency, duration) considering their organisational processes, pragmatic considerations such as resources and staff availability.

The final pet intervention protocol (Supplementary material 1) involved up to 30 min of pet robot intervention, 3 times weekly, over 6-weeks between February to July 2025. At the outset of each session, staff delivering the intervention would bring two types of robotic pets (i.e., cat, dog or bird) and provide the opportunity to choose their preferred robot. The intervention period was decided in consultation with key staff, and is in line with previous pet robot interventions (Pu et al., 2020; Liang et al., 2017; Koh et al., 2021). This protocol allowed for flexibility for staff to decide on whether interventions should be actively facilitated or unfacilitated (i.e., residents to use pet robots without supervision) involvement. This enabled contextual adaptability by allowing staff to use the intervention at different times of the day based on residents’ needs, care staff’s availability and dynamic work environments in RACFs - which has been widely reported as barriers to sustainable implementation of pet robots in dementia care (Koh et al., 2021; Koh et al., 2022b).

### Data collection and analysis

At the end of the intervention period, residents, their family members and recreational officers involved in delivering the intervention were invited to take part in individual, face-to-face semi-structured interviews based on a descriptive qualitative approach. Eight residents with dementia had consented to participate, however one had passed away before the intervention commenced. A robotic pet was brought to each interview session as a visual prompt, particularly for residents with dementia, to facilitate discussions. An interview guide (Box 1) was used to guide the interviews which were conducted in-person and audiorecorded.

Box 1 **Semi-structured interview guide.**

Residents with dementia

1. What do you think about the robotic pet?
2. What does the robotic pet do for you?
3. What do you not like about the robotic pet?
4. What do you think about how frequent you get to spend time with it?
5. Do you prefer someone to be with you when you spend time with the pet?
6. What do you think about sharing the pet with other residents?

Staff and family members

1. What do you think about the robotic pet?
2. What was your experience of using the robotic pet?
3. What impact do you think it had on residents, if any?
4. What impact did it have on you, if any?
5. What are your main concerns about using robotic pets?
6. What other supports do you think should be in place to support staff to introduce pet robots for dementia care

Fieldnotes were taken during the interviews. Photographs of residents’ interaction with robotic pets were also taken if permission was provided. Questions were focused on understanding the experiences of the impact of using or implementing the pet robots. Data from the semi-structured interviews were transcribed and analysed using inductive qualitative content analysis as described by Elo and Kyngäs (2008). NVivo15 was used for data management and organisation; this also added rigour to the analysis process by providing an audit trail. First, WQK immersed herself with the data by reading and rereading the transcripts, taking notes and annotated memos to make sense of the data. Next, WQK read through each transcript again and started to assign open codes. At this stage, preliminary categories were also generated concurrently. Thereafter, the categories were re-organised and grouped into categories. Finally, these categories were reviewed and interpreted to generate final themes. The Standards for Reporting Qualitative Research (SRQR) (O’Brien et al., 2014) was used to guide the reporting of qualitative findings.

## Findings

Eight participants including five residents, one family member and two staff (recreational officers) participated in the interviews. Participants’ demographics are presented in Table 2. While both facilitated and unfacilitated interventions were available for staff to choose from, all pet robot interventions that were delivered were facilitated.

**Table 2:** Participants’ demographics.

Pseudonym	Gender	Stakeholder group	Age/age group
Carol	Female	Family member (daughter)	Not provided
Bob	Male	Resident with dementia	88
Ellie	Female	Resident with dementia	99
Grace	Female	Resident with dementia	90
Marie	Female	Resident with dementia	86
Tina	Female	Resident with dementia	90
Alice	Female	RACF recreational officer, 6–8 years of experience	Not provided
Katie	Female	RACF recreational officer, 10 + years of experience	Not provided

Four key themes were generated, including (1) perceptions of the attributes of pet robots, (2) opening doors – facilitating connections and participation, (3) integrating pet robots into routine care, and (4) moving forward - maximising impact and sustainability. Supplementary material 2 provides a summary of the themes and exemplar quotes.

### Theme 1: perceptions of the attributes of pet robots

This theme describes participants’ perceptions of their interactions with robotic pets. All participants expressed positive sentiments about robotic pets. While three types of robotic pets (dog, cat and bird) were provided as options for residents, only the JfA cats and dogs were chosen by participants; none chose to engage with the JfA bird. All residents have had experiences of owning domestic pets including cats and dogs. Accordingly, the design of JfA pets as cats and dogs were described as more relatable to residents than ones designed as less familiar animals:

*“I had seen the seal before, that was many moons ago…, it was really just sort of put into a cupboard because it didn't relate to anybody… always through our admission process, it was a lot of things that people were missing when they came to aged care with their animals. It was always a cat and dog… when these came about, and they're quite life-like for the residents… they could relate a lot better to them than the big seals” (Katie, recreational officer)*

While a few residents have indicated preferences for real cats or dogs based on their past experiences, some residents did not select a robotic pet based on this. For instance, a resident who had a clear preference for pet dogs indicated a stronger preference for the robotic cat: *“I like this one (JfA cat) but usually I prefer dogs” (Ellie, R4, resident aged 90)*. All participants expressed that they enjoyed the pet robots’ appearance such as their colour and fur coat. For example, one resident expressed *“Oh they (JfA pets) are lovely, they’re beautiful” (Grace, resident aged 86)*. However, interactions with robotic pets were limited in some instances due to their design, or due to residents’ physical or sensory abilities. One resident with hearing impairment was not able to hear the meowing and barking sounds of the JfA pet. Given that the JfA dogs assumed a seated position, staff felt that it was harder for residents to hold it as compared to the JfA cats, which were in a lying position. One staff shared that the weight of the JfA pets was unmanageable for some residents, *“the size (of the JfA dog and cat) is so big… they can’t hold it… it’s heavy for them” (Alice, recreational officer)*. This sentiment also emerged in an interview with one of the residents, who shared about difficulties interacting with the robotic pet due to upper limb movement limitations:

*“When I put my hand there (gesturing reaching forward for a robotic pet placed on the wheelie walker in front of her) to pat it, it hurts my shoulder” (Tina, resident aged 90)*

### Theme 2: opening doors – facilitating connection and participation

This theme describes participants’ perceptions of how robotic pets provided points of connection and facilitated participation. Both the family member and staff participants described the JfA pets as providing opportunities for them as caregivers to connect with residents who were more withdrawn, such as those who spent more time in their own rooms and did not engage in group activities:

*“In the room, they were stuck on their own. So I thought it was a good thing… (the robotic) pets were a good introduction… allow us to go into the room and then we could sit down and have a conversation… it was a nice thing to use to get into some rooms where they (residents) wouldn't allow you normally to go into”*

*(Katie, recreational officer)*

**Figure 1:**
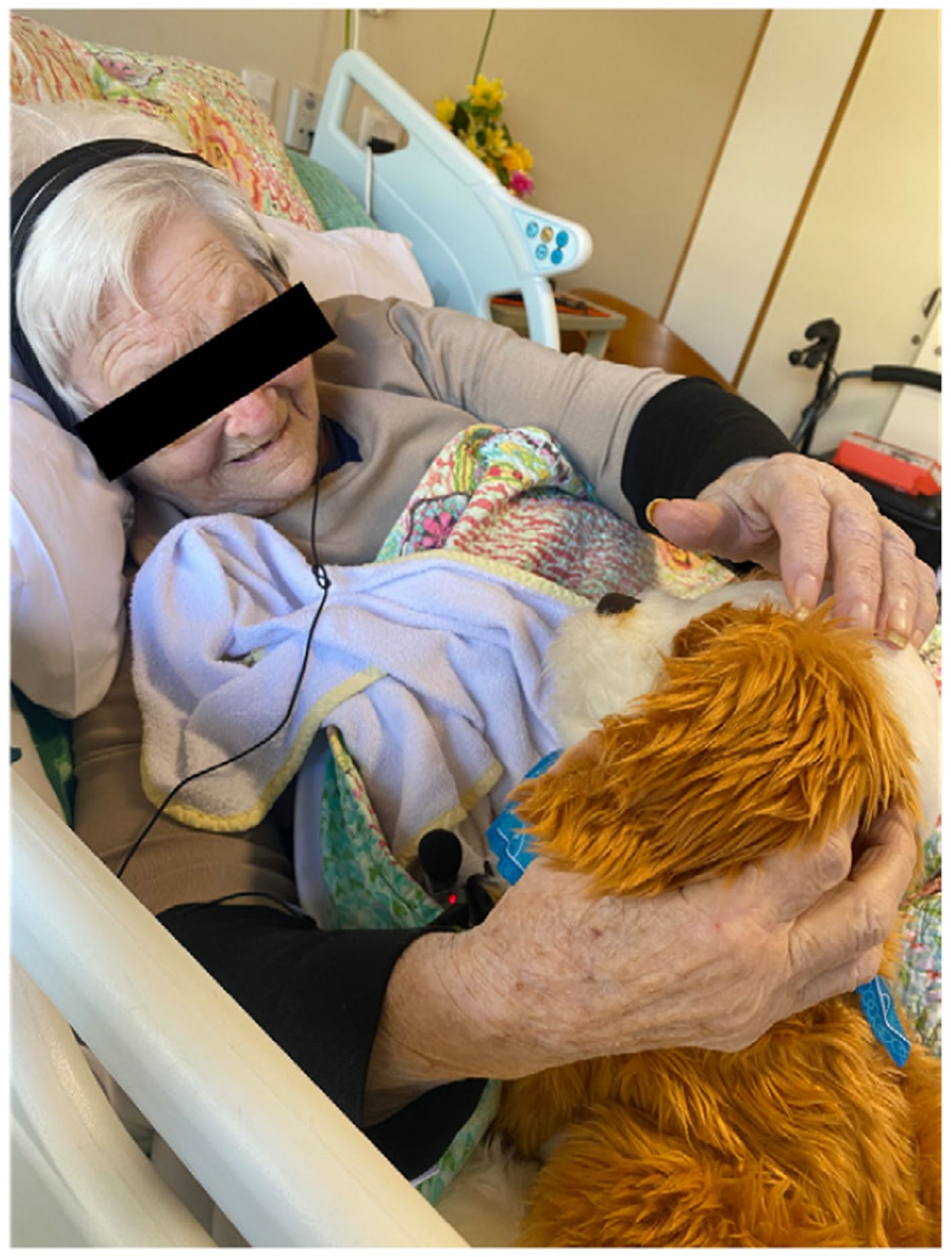
Residents interacting with robotic pets.

By the same token, they described residents’ connections to the robotic pets which involved residents interacting with them, such as talking to the robotic pet, asking questions about them such as their names, petting or holding them (Figure 1). Some residents engaged with the robotic pets to an extent that influenced their routines and participation in other activities within the facility.

One staff described an example in which engagement with the robotic pet calmed a resident, who is described as being a ‘non-stop walker’ who wanders around the facility. Engaging with the JfA pets enabled her to relax with it on the couch:

*“she's a non-stop walker… her walking is fast… (we were) just sitting next to her on the couch with the (JfA) dog or cats, (she is) just completely resting. I remember two or three times, she ended up… having a nap” (Alice, recreational officer)*

A family member described her mother, Carol, as someone who was withdrawn despite being a resident of the facility for years. Carol connected with the robotic dogs, which provided her with comfort and facilitated engagement in other activities:

*“She was not going out there (into facility’s common areas) before then. Couple of times she did, she’d do it for a week, then lose interest and stay in her room. And now she’s been out there for a few months now… she had a need, that dog was meeting it… she is a bit more relaxed… She never thought about this place as a home, till the dog came along” (Grace, daughter of a resident)*

Consequently, she expressed that the robotic pets “*lets her off the hook*”, and provided her with a greater sense of relief during visitations where she felt less pressured to support her mother. Another resident expressed feeling ‘lucky’ to interact with the JfA pet, sharing: *“it almost feels a part of me. And that would make a lot of other individual… they’re going to feel much better” (Bob, R6, resident aged 88)*.

While other residents also engaged with the robotic pets, they preferred a shorter engagement with the robotic pets. While not all residents were able to provide reasons, one expressed that she gets ‘*bored easily*’. Staff expressed the intervention duration varied between residents, ranging from 5 min to 20 min each time. They explained that robotic pets served different purposes for different residents, which could explain varying degrees of engagement and impact:

*“…it depends on the resident that you’re using it (robotic pet) for… (for some, it) kept them calm. The other ones, it was more of a distraction from doing something else… it depends on the resident, it really does… (some residents) could only sit still and focus for that 5-10 minutes. And the other ones that did work a lot longer for. It wasn't more of a distraction, it was a comfort for them” (Katie, recreational officer)*

### Theme 3: integrating pet robots into routine dementia care

This theme describes staff experiences of integrating pet robots into their routine dementia care and impact on caregiving responsibilities. Staff who delivered the intervention shared about their decision making process on what time of the day they decide to use robotic pets. Considerations centred around their personal work schedules where they tried to ‘*mould it into*’ their work routine, and their understanding of residents’ routines:

*“… (in the) afternoon most of them are in bed…. (but afternoons are) the best thing for (name of resident) who needs a rest, to help her sit down, and you can see it…” (Alice, recreational officer)*

Staff perceived that having robotic pets was another ‘tool’ that staff could use to support residents with dementia. As compared to other existing interventions or activities, such as quizzes, painting or virtual reality, robotic pets offered residents a sensory, ‘natural’ experience that were reminiscent of their past experiences. Staff participants expressed that the intervention frequency of three times weekly did not alter their work routines, as they (recreational officers) had more stable staffing and less staffing changes as compared to other disciplines like nursing. Having the robotic pet intervention overseen by one identified discipline was identified as an important factor influencing the sustainability of interventions. Participants also explained that the subcontexts within the facility also influenced how likely staff from other discplines were to use the robotic pets as part of their routine care. The dementia unit was described to be a “less busy” environment than the general unit; and staff working in the dementia unit could dedicate more time to support residents with dementia:

*“… down in the (dementia) unit… it's not as busy, but it's more behaviours and that. So you have got a bit more time to have that one-on-one and sit down. But here (general unit)… you've got 40 of them running around and they're just go, go, go… down there they've got a bit more time to sit and do the one-on-one… So it benefits down there (dementia unit). Yeah. I can see it really good… They're not falling… some of them fall asleep with that (robotic pets), especially the cats” (Katie, recreational officer)*

The impact of pet robots in calming residents are described as being perceived positively by staff, including those who were not directly involved in delivering the intervention. Alice, an aged care staff expressed that having a resident calm down and have reduced wandering behaviours was the *“best thing for the staff, the nurses because… because she has a very,very high risk of falling and she had (fallen) a lot of times” (Alice, recreational officer).*

### Theme 4: moving forward – maximising impact and sustainability

This theme describes participants experiences of the sustainability of the intervention, and how efforts to increase their impact and sustainability could be further enhanced. At the time of the interview, which occurred between one to three months after the intervention period (due to participants’ and the research team’s availability), pet robots have been integrated into the routine care. Nevertheless, staff described efforts that could be undertaken to enhance implementation efforts, and to maximise their impact and sustainability. These should include a greater extent of staff involvement and buy-in to better understand the impact of pet robots as a non-pharmacological intervention:

*“But then it was only us that go, oh, they're agitated, okay… let's give them the pet (robot). Whereas other (staff could) be like, ‘oh, I don't know, let's medicate’. Instead of using medication, you could just give them a (robot) cat and they would've been fine. Not everyone was fully on board (and were) able to take that time… everyone's busy… (but if) everyone’s sort of involved in that process… they would've seen it, how beneficial they (robotic pets) were. Some (staff) were really good with that. But if we wanted to see something, like amazing difference in someone, everyone needs to be involved” (Katie, recreational officer)*

Participants also expressed that better communication between staff in terms of knowing where the robotic pets are is essential, as not everyone knew which staff or residents had the robotic pets when they needed it. To ensure sustainability, staff also expressed that they have to be placed in visible and readily accessible places instead of closed cupboards. This strategy that was put into place by staff had been described to contribute to continued use of robotic pets, in stimulating interest among other residents and family members:

*“They (robotic pets) just need to be out there… the one good thing that we got was we had them out there and they (led to) curiosity questions (from residents and family members). It was something for them to voluntarily go and have a look and then start petting and instead of going, oh, such and such is upset. Let's get this out of the cupboard” (Katie, recreational officer)*

Sustainability also had to be considered in regards to the availability and accessibility for residents. A family member had expressed her initial concerns about the availability of pet robots at the end of research and described a sense of relief when she was informed by staff that the robotic pets will continue to be available:

“*They can’t take them away. How can you give something that changes (a resident) and take it away?’ I was going to… protest, I was thinking that you know? But of course, you’re not taking it away, it’s not duty of care to do that” (Carol, family member of a resident)*

Similarly, staff expressed concerns about the durability of the JfA pets and their impact on residents. Accordingly, staff participants advocated for the need for more pet robots within the facility. This is also in consideration of infection control, where sharing of robotic pets between residents was seen as undesirable to prevent potential transmission of infections. Finally, volunteers were also suggested as means to deliver robotic pet interventions, as residents often build good rapport with volunteers and would be well-placed to support intervention delivery:

*“Volunteers would just love it (robotic pets). And it's, they've got a different rapport with the residents than what we have… they see the volunteers as they're not staffing, but somebody from outside that has no, opinion that nothing to do with their medications or their stay. They're just like a neighbour, someone really friendly… plus they've got a bit more time (with residents) because they've got allocated time” (Katie, recreational officer)*

## Discussion

This research aimed to evaluate the impact and implementation of a 6-week pet robot intervention to support residents with dementia in a residential aged care facility. The intervention and implementation plans were co-developed in consultation with stakeholders from the facility. Post-intervention interviews revealed that the pet robots’ attributes were perceived positively. Specifically, their design as familiar domestic animals and the range of pets available for residents to choose from were well regarded. Residents chose the robotic cat and dog, but none chose the robotic bird as their preferred pet robot. While participants did not explain why, this might be in part due to their past experiences with pets. Participants discussed their familiarity with dogs and cats during the interviews, however none described experiences with birds. Robotic pets provided participants with a point of connection and residents had different levels of engagement; for some residents, the robotic pets facilitated participation in activities and routines and provided calming effects; others were not able to sustain engagement. Nevertheless, given that interventions were facilitated by staff, it is difficult to ascertain whether the positive impacts resulted from facilitation or interactions with the pet robots (Moyle et al., 2017). While each session’s intervention duration varied based on residents’ engagement, delivering the robot intervention three times weekly was feasible as staff were able to work this into their schedules and residents’ routines. To maximise the impact and sustainability of pet robot interventions, a greater extent and depth of staff involvement was regarded as necessary. The accessibility and availability of pet robots were also highlighted as essential.

Our participants’ perceptions of pet robots and the intervention echoed findings from previous studies; people living with dementia positively perceived the robots’ lifelikeness and familiar design as dogs and cats as being relatable (Bradwell et al., 2021). Nevertheless, the robotic cat was used more frequently by participants likely due to its shape as being easier and more intuitive to hold. Participants’ experiences of the impact on mood and social connection also resonates with current research evidence (Moyle et al., 2017; Pu et al., 2020; Bradwell et al., 2021). Residents with dementia in this study had varying levels of engagement with robotic pets. This echoes findings from a qualitative evidence synthesis, which also noted that people with dementia had varying responses to robotic pets (Scerri et al., 2021), which are ‘not for everybody’ (Birks et al., 2016). While pet ownership has been suggested as a potential indicator as to whether an individual might take to robotic pets (Dementia Support Australia., 2022, Scerri et al., 2021), our findings suggest otherwise; despite all residents having past experiences with domestic animals, their levels of engagement with robotic pets varied. Likewise, personal experiences with pets may not provide a clear indication of their preferred pet robot - for instance, a resident who had a clear preference for pet dogs preferred the robotic cat. Previous research also showed that non-pet owners can still benefit from robotic pets (Pike et al., 2021). These suggest that while individuals’ experiences with pets may provide a starting point to gauge whether they may engage with robotic pets, it would still be important to provide residents with equal opportunities for engagement regardless of their previous experience.

Previous studies have identified several barriers to integrating robotic pets in routine dementia care, which included high costs, increased staff workload, staff shortages, and a lack of human and time resources to facilitate the use of robotic pets (Sung et al., 2015; Moyle et al., 2017; Koh et al., 2022b). To overcome prospective barriers, the intervention and implementation plans in this study was co-developed with staff from the RACF; the facility’s readiness to implement robotic pets were ascertained prior this study, education sessions were conducted, and the intervention was designed to allow for flexibility based on staff schedules and their familiarity with residents’ routines and preferences. While there was no formal, quantitative evaluation of the implementation given this study’s exploratory nature, qualitative interviews show that robotic pets sustained beyond the 6-week intervention period, suggesting that the co-developed plans supported the integration of the intervention into routine care. The sustained use of robotic pets may also be attributed to their positive impact on residents; Scerri and colleagues (2020) found that when staff understood and saw the positive impacts of robotic pets, the implementation is often perceived positively. Visible placement was another strategy that was employed by staff in this study, who have chosen to place their robotic pets in a visible, accessible place at the RACF’s common living space. Niemelä et al. (2016) identified that the visibility and accessibility of robots affected their routine use. Likewise, Felding et al. (2024) found that when pet robots were shelved or placed in a locked office, they became ‘forgotten’ and unused.

While initial education sessions were conducted to facilitate buy-in from staff within the facility, staff with primary involvement in delivering the intervention had expressed that not all staff members were on board. This has partly been attributed to differences in staffing stability and responsibilities across different disciplines. A qualitative study involving 22 RACF staff found that some staff or specific disciplines such as activity coordinators (also known as recreation officers or diversional therapists in other countries), may have more flexibility within their routines to allow for the use of pet robots as part of their work routine (Koh et al., 2022b). In contrast, some disciplines with staff shortages or higher staff turnover may have lesser flexibility and could encounter more implementation constraints. Staff participants have also noted that there is a lack of shared understanding across all staff members of the impact of pet robot intervention as a non-pharmacological alternative to psychotropic medication use, which led to an implicit reliance on specific staff to implement robotic pets. Generating staff buy-in has been highlighted as an important implementation determinant (Greenhalgh et al., 2004), and implementation barriers relating to staff buy-in has been reported for other interventions in RACF contexts (Seshadri et al., 2021; Devi et al., 2023). To encourage staff buy-in, previous research suggests that ongoing education was found to be more useful than one-off education sessions, especially considering staff turnover (Seshadri et al., 2021). Another strategy that has been found to be critical includes explicit leadership support, such as advocacy and presentation of a clear vision of care (Quasdorf and Bartholomeyczik, 2019) as supported by technology like pet robots.

To increase the sustainability of pet robot interventions, staff participants have also suggested tapping into the use of volunteers for pet robot intervention delivery to overcome staff resources limitations. With adequate training, information and support from staff, volunteer led delivery of activities for people with dementia in RACFs have been found to be feasible (Van der Ploeg et al., 2014; Hurst et al., 2019). To the best of our knowledge, there has been no research done on volunteer-led pet robot intervention delivery. Given that pet robot interventions require human facilitation, volunteers may represent a resource that could be leveraged to supplement the aged care workforce to improve intervention sustainability and dementia care quality.

### Strengths and limitations

A key strength of this study lies in its collaborative nature, where the pet robot intervention and implementation plan were developed in consultation with key RACF staff to ensure sufficient contextual considerations. Likewise, the implementation of the intervention was driven by RACF staff rather than research resources. As such, findings from this study reflect real-world experiences, impact and barriers of implementing pet robots in RACFs for dementia care. We ensured rigour and trustworthiness by maintaining a clear audit trail through NVivo15 as a data management tool, providing clear description of participants’ characteristics and to support readers’ interpretation of the findings, and providing clear verbatim quotes to support our analysis. However, we did not collect information about the type of dementia and dementia severity, which limits contextual interpretation. This study is primarily limited by its small sample size and exploratory nature. Only one family member and two staff consented to participate in the interviews post-intervention. Due to a lack of funding and other pragmatic resources (e.g., human resources), we were not able to collect quantitative data on preliminary effectiveness outcomes and conduct a formal evaluation of the implementation strategies used, which limits the generalisability of findings. The intervention protocol was intended to be adaptable to contextual demands in residential care contexts (i.e., 3 times a week, for up to 30 min each session, which can be either facilitated or unfacilitated), to accommodate staff and residents’ availability and preferences. While this frequency was agreed upon with staff from the outset to accommodate contextual demands, a lack a ‘standard’ intervention protocol is another study limitation that should be acknowledged. The data was analysed by a single researcher (WQK) which could be a limitation. However, preliminary themes and final themes were discussed with a co-researcher (NG) for sense checking to allow for a more holistic interpretation of the data. Likewise, final themes were shared with the wider research team, who had the opportunity to provide feedback on the themes and data interpretation. Finally, given that interventions were facilitated, it is difficult to ascertain whether the positive impacts resulted from staff facilitation or interactions with the pet robots (Moyle et al., 2017).

## Conclusion

In this study, we described the co-development and implementation of a 6-week pet robot intervention with staff from a RACF for dementia care. Post-intervention interviews revealed that the pet robots’ attributes were perceived positively. Robotic pets provided participants with a point of connection, however residents had varying levels of engagement with the robotic pets. The 6-week intervention was feasible, and intervention sustainability continued beyond the intervention period. To maximise intervention impact and sustainability of pet robot interventions, a greater extent and depth of staff involvement was seen as necessary. The findings from this study provide insights into strategies that might increase the routinisation of pet robots in dementia care; future studies could consider further refining, enhancing and formally evaluating these strategies. Future research should consider evaluating the use of volunteers as a resource to supplement staffing limitations in RACFs and to increase the sustainability of pet robots and other technology-based interventions in dementia care.

## Data Availability

The datasets presented in this article are not readily available because raw data from the qualitative interviews are not made publicly available to protect participants’ privacy and anonymity. Anonymised transcripts may be made available from the corresponding author upon reasonable request. Requests to access the datasets should be directed to Wei Qi Koh, weiqi.koh@uq.edu.au.
