# Supplementary material for: Evaluating a co-developed pet robot intervention and implementation for residents with dementia in long-term care
Source: Front Dement. 2026 Apr 17;5:1791588. doi: 10.3389/frdem.2026.1791588 (PMC13133679; doi:10.3389/frdem.2026.1791588)
Supplement: Supplementary file 1 [file Supplementary_file_1.docx]

**Supplementary File 1: Intervention protocol**

**1.1 – Facilitated intervention**

| Introduce robotic pets to residents | Bring all robotic pets (dogs, cats, bird) to residents for first session, and introduce them to residents. Encourage resident to choose the robotic pet of their preference. For subsequent sessions, bring the robotic pet of the individual’s preference. |
| --- | --- |
| Robot interaction | Observe resident’s response to the robot, and encourage social interactions with the robotic pet and encourage conversations, e.g., asking ‘what do you want to name (robotic pet)? |
| Ceasing pet robot interaction | After up to 30 minutes of interaction, return to the participant to collect the robotic pet, and arrange a next session for robotic pet interaction. |

**1.2 – Unfacilitated intervention**

| Introduce robotic pets to residents | For first session:  Bring all robotic pets (dogs, cats, bird) to residents, and introduce them to residents for first session. Encourage resident to choose the robotic pet of their preference. For subsequent sessions, bring the robotic pet of the individual’s preference. Observe resident’s response to the robot. If resident amenable to robotic pet or does not have a negative response, let resident interact with it. |
| --- | --- |
| Robot interaction | Observe robotic pet interaction from a distance |
| Ceasing pet robot interaction | After up to 30 minutes of interaction, return to the participant to collect the robotic pet, and arrange a next session for robotic pet interaction. |

After robot pet interactions with each resident, the robotic pets can be cleaned (wiped down) with disinfectant
