# Supplementary material for: Evaluating a co-developed pet robot intervention and implementation for residents with dementia in long-term care
Source: Front Dement. 2026 Apr 17;5:1791588. doi: 10.3389/frdem.2026.1791588 (PMC13133679; doi:10.3389/frdem.2026.1791588)
Supplement: Supplementary file 2 [file Supplementary_file_2.docx]

**Supplementary Material 2 - Summary of the themes and exemplar quotes**

| **Theme** | **Codes** | **Exemplar quotes** |
| --- | --- | --- |
| Theme 1: Perceptions of the attributes of pet robots | Familiar pets are more relatable | “I just had the seal before yet, that was many moons ago..., it was really just sort of put into a cupboard because it didn't relate to anybody… always through our admission process, it was a lot of things that people were missing when they came to aged care with their animals. It was always a cat and dog… when these came about, and they're quite life-like for the residents… they could relate a lot better to them than the big seals” (Katie, recreational officer) |
|  | Enjoying pet robots’ appearances | “Oh they (JfA pets) are lovely, they’re beautiful” (Grace, resident aged 86) |
|  | Features limiting interactions | “When I put my hand there (gesturing reaching forward for a robotic pet placed on the wheelie walker in front of her) to pat it, it hurts my shoulder” (Tina, R3, resident aged 90) |
|  | Impact of previous pet experiences | “I like this one (JfA cat) but usually I prefer dogs” (Ellie, resident aged 90) |
| Theme 2: Opening doors – facilitating connection and participation | “Lets me off the hook” | “… (the robotic dog is) really good for me as it lets me off the hook, you know?” (Grace, daughter of a resident) |
|  | Calms residents who are restless | “she's a non-stop walker… her walking is fast… (we were) just sitting next to her on the couch with the (JfA) dog or cats, (she is) just completely resting. I remember two or three times, she ended up.... having a nap” (Alice, recreational officer) |
|  | Increased participation in activities | “She was not going out there (into facility’s common areas)) before then. Couple of times she did, she’d do it for a week, then lose interest and stay in her room. And now she’s been out there for a few months now… she had a need, that dog was meeting it… she is a bit more relaxed… She never thought about this place as a home, till the dog came along.” (Grace, daughter of a resident) |
|  | Feeling happy about intervention impact | “I go home happier now because I know that she is more enriched than she used to be” (Grace, daughter of a resident)  “It almost feels a part of me. And that would make a lot of other individual… they’re going to feel much better” (Bob, resident aged 88) |
|  | Feeling bored when interacting with robot | “There’s nothing I don’t like about her (robotic pet). I just get bored easily” (Marie, resident with dementia, aged 86) |
|  | Encouraged interactions and connections | “… especially the cat, she loves it… it's like a good connection” (Alice, recreational officer) |
|  | Intervention duration and frequency depends on residents | “…it depends on the resident that you’re using it (robotic pet) for… (for some, it) kept them calm. The other ones, it was more of a distraction from doing something else… it depends on the resident, it really does… (some residents) could only sit still and focus for that 5-10 minutes. And the other ones that did work a lot longer for. It wasn't more of a distraction, it was a comfort for them”  (Katie, recreational officer) |
| Theme 3: Integrating pet robots into routine dementia care | Dedicated time in dementia units | “… down in the (dementia) unit… it's not as busy, but it's more behaviours and that. So you have got a bit more time to have that one-on-one and sit down. But here (general unit)… you've got 40 of them running around and they're just go, go, go… down there they've got a bit more time to sit and do the one-on-one... So it benefits down there (dementia unit). Yeah. I can see it really good… They're not falling… some of them fall asleep with that (robotic pets), especially the cats” (Katie, recreational officer) |
|  | Considering when to use robotic pets | “I sort of tried to mould it (pet robot intervention) into what we were sort of doing (within work routines)” (Katie, recreational officer) |
|  | Another ‘tool’ that staff can use | “In any way they're just a different... just something that we can use. So it's another tool now that we could use that we know, okay, they really, they really respond well to this. So, you know, instead of going, come on, you like painting or something, and you think, well, I don't want to do painting… (robotic pets) are just very sensory” (Katie, recreational officer) |
|  | Relieve work or workload | “… (in the) afternoon most of them are in bed…. (but afternoons are) the best thing for (name of resident) who needs a rest, to help her sit down, and you can see it…” (Alice, recreational officer) |
| Theme 4: Moving forward – maximising impact and sustainability | Continued use of robotic pets | “…now they've sort of taken ownership (with some residents after the intervention period” (Katie, recreational officer) |
|  | Displaying pet robots publicly stimulated interest | “They (robotic pets) just need to be out there… the one good thing that we got was we had them out there and they (led to) curiosity questions (from residents and family members). It was something for them to voluntarily go and have a look and then start petting and instead of going, oh, such and such is upset. Let's get this out of the cupboard” (Katie, recreational officer) |
|  | Equal and continued access to pet robots | “They can’t take them away. How can you give something that changes (a resident) and take it away?’ I was going to… protest, I was thinking that you know? But of course, you’re not taking it away, it’s not duty of care to do that”. (Carol, family member of a resident) |
|  | Need for more organisation wide involvement | “… everyone needs to be involved a bit more. I felt it was a bit more on probably our staffing. Which is fine. But then it was only us that go, oh, they're agitated. Okay, well, they're upset. Let's give them the pet where, you know, other disciplines to be like, oh, I don't know, let's medicate. Instead of using medication, you could just give them a cat. Yeah. And they would've been fine” (Katie, recreational officer) |
|  | Volunteers could support pet robot use | “Volunteers would just love it (robotic pets). And it's, they've got a different rapport with the residents than what we have… they see the volunteers as they're not staffing, but somebody from outside that has no, opinion that nothing to do with their medications or their stay. They're just like a neighbour, someone really friendly… plus they've got a bit more time (with residents) because they've got allocated time” (Katie, recreational officer) |
